# MxA Is a Novel Regulator of Endosome-Associated Transcriptional Signaling by Bone Morphogenetic Proteins 4 and 9 (BMP4 and BMP9)

**DOI:** 10.1371/journal.pone.0166382

**Published:** 2016-11-22

**Authors:** Huijuan Yuan, Pravin B. Sehgal

**Affiliations:** 1 Department of. Cell Biology & Anatomy, New York Medical College, Valhalla, New York, United States of America; 2 Department of. Cell Biology & Anatomy, and Department of Medicine, New York Medical College, Valhalla, New York, United States of America; University of Illinois at Chicago, UNITED STATES

## Abstract

There is confusion about the role that IFN-α plays in the pathogenesis of pulmonary arterial hypertension (PAH) with different investigators reporting a causative or a protective role. There is now clear evidence in PAH pathogenesis for the involvement of BMP4 and BMP9 signaling, and its disruption by mutations in BMPR2. In the present study, we investigated MxA, an IFN-α-inducible cytoplasmic dynamin-family GTPase for effects on BMP4/9 signaling, including in the presence of PAH-disease-associated mutants of BMPR2. In human pulmonary arterial endothelial cells (HPAECs), IFN-α-induced endogenous as well as exogenously expressed MxA was associated with endosomes that aligned alongside microtubules and tubules of the endoplasmic reticulum (ER). Moreover, IFN-α and MxA stimulated basal and BMP4/9 signaling to a Smad1/5/8-responsive pBRE-Luc reporter. In HEK293T cells, immunoelectron microscopy confirmed the association of MxA with endosomes, and immunofluorescence methods showed these to be positive for early endosome markers (early endosomal antigen 1, clathrin light chain and Rab5) and RSmad1/5/8. Functionally, using different genetic and inhibitor approaches, we observed that clathrin-mediated endocytosis enhanced and caveolin-mediated endocytosis inhibited the transcriptional response to BMP4 and BMP9. MxA produced a further 3-4-fold enhancement of the BMP-induced response in a clathrin-endocytosis dependent manner. The microtubule inhibitor nocodazole and stabilizer paclitaxel respectively attenuated and enhanced the effect of MxA, implicating microtubule integrity in this process. MxA enhanced BMP-induced signaling in the presence of wild-type BMPR2, and partially rescued signaling from some PAH-disease-associated BMPR2 mutants. Taken together, the data identify MxA as a novel stimulator of BMP4 and BMP9 transcriptional signaling, and suggest it to be a candidate IFN-α-inducible mechanism that might have a protective role against development of PAH and other vascular diseases.

## Introduction

Idiopathic pulmonary arterial hypertension (PAH) is a devastating disease with high morbidity and mortality affecting young women 2-4-fold more frequently compared to men (median age at onset in women is in the third decade; in men it is in the fourth decade) [1,2,3]. In this disease, there is a decrease in the lumen of precapillary pulmonary arterial segments due to vascular remodeling characterized by enlarged tunica media leading to the typical onion-skin or plexiform lesions [1,2,3]. Hereditary PAH comprises kindreds with haploinsufficiency of or autosomally dominant mutations in the *bone morphogenetic receptor protein 2* (*BMPR2*) gene [1,2,3]. A consequence of these mutations in BMPR2 is a decrease in transcriptionally productive signaling by the bone morphogenetic proteins (BMPs), allowing greater proliferation of vascular cells. BMP signaling typically inhibits vascular proliferation; indeed studies in the last decade have identified BMP4 and BMP9 to be of special relevance in maintaining vascular endothelial and smooth muscle cells in a state of quiescence [4,5]. Thus, disruption of BMP4 and/or BMP9 signaling is thought to be a contributory factor in reversing this quiescent state culminating in vascular cell proliferation and PAH. Additionally, acquired PAH (without underlying mutations in *BMPR2*) has resulted from the ingestion of anorexigenic drugs and plant products (e.g. rape seed oil), and parasitic infections (e.g. schistosomiasis) with pathogenesis thought to include underlying inflammation in pulmonary arterial segments accompanied by local cytokine production [3,6]. Indeed, an unusual example of acquired PAH occurs in patients, mainly women, who have been administered Type I interferons (IFNs). A small subset (approximately 0.5%) of patients, mainly women, administered IFN-α or β for chronic myelogenous leukemia, multiple sclerosis or other conditions developed PAH, which could be reversed in some but not all patients by cessation of the IFN therapy [7,8,9].

An understanding of the underlying mechanisms of this pathogenesis has been confusing. George *et al* [10] reported that male *IFNAR1-/-* mice were protected from development of PAH upon exposure to chronic hypoxia. Specifically, George *et al* [10] concluded that Type I IFNs mediate PAH. In contrast, Bauer *et al* [11] reported that IFN-α2b administration inhibited development of PAH in male rats exposed to the inhibitor SU5416 and chronic hypoxia or in male mice exposed to chronic hypoxia alone. Moreover, *IFNAR1-/-* mice developed PAH equivalent to that observed in *wt* mice after chronic hypoxia, and were not protected by IFN-α administration. Thus, Bauer *et al* [11] provided data showing that IFN-α was protective in hypoxic PAH in male mice. Bauer and colleagues noted the contrast between their data, especially in hypoxic *IFNAR1-/-* male mice, and those of George *et al* [10], and expressed puzzlement about the underlying mechanisms that might lead to contrasting outcomes. In attempting to understand these contrasting observations, we focused on a protein long-known to be specifically induced by Type I IFNs–the myxovirus resistance protein A (MxA)–and whether this protein might affect signaling in response to BMP4 and BMP9 [12,13,14].

MxA and related family members (MxA and MxB in humans; Mx1 and Mx2 in mice) are typically upregulated 10-100-fold by Type I (IFN-α species and IFN-β) and Type III (IFN-λ species) IFNs but not by Type II (IFN-γ) IFN [12,13,14]. The 70-kDa human MxA protein is a cytoplasmic dynamin-family atlastin-like GTPase and is a mediator of the broad-spectrum antiviral activity of IFNs [12,13,14]. At the biochemical level, MxA molecules oligomerise into dimers and multimeric rings, and bind intracellular membranes causing membrane bending and tubulation [15,16]. In intact cells, MxA has been reported to increase caveolar endocytosis and enhance IL-6/STAT3 signaling [17]. In as much as it is well established that signaling initiated at the plasma membrane by several cytokines and growth factors, including transforming growth factor β (TGF-β), BMP2, and IL-6, transits the cytoplasm along membrane-associated endocytic pathways to generate a transcriptional response [17,18,19], we investigated the possibility that the IFN-inducible membrane-bioactive MxA might affect BMP4 and BMP9 signaling, and thus participate in IFN-mediated alterations in PAH pathogenesis.

In the present study we have investigated the organellar association of IFN-α-induced endogenous MxA in human pulmonary arterial endothelial cells (HPAECs), as well as that of exogenously expressed MxA in HPAECs and HEK293T cells. With the discovery that MxA in such cells was associated with early endosomes (the MxA-endosomes) which were, in turn, associated with microtubules and ER tubules as scaffolds [20,21,22,23,24], we investigated whether IFN-α and MxA affected productive transcriptional signaling by BMP4 or BMP9. This provided us with a model system to test the functional consequences of endosome trafficking in association with microtubules and ER tubules suggested by the scaffolding hypothesis of Voeltz and colleagues [24]. We also investigated whether MxA might rescue the inhibitory effects of PAH-disease causing mutants of BMPR2 on BMP4/9 signaling. Overall, the discovery of MxA endosomes and their contribution towards stimulating BMP4/9 signaling identifies a novel candidate mechanism by which Type I IFNs could be protective in the pathogenesis of vascular disease.

## Results

### MxA-endosomes in HPAECs lie alongside ER tubules and microtubules

**Fig 1:**
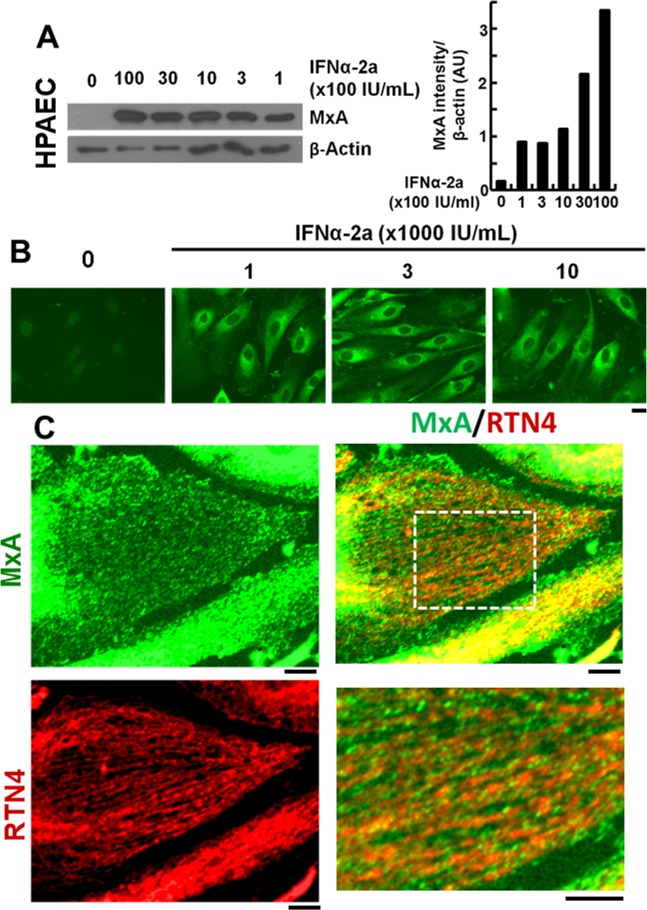
Endogenous MxA in HPAECs localizes to endosomes that lie alongside ER tubules. HPAEC cultures were exposed to IFN-α for 16 hr at the indicated concentrations. Panel A illustrates Western blots for MxA and β-actin of whole cell extracts (80 μg protein/lane); quantitation of the blot shown in terms of MxA induction normalized to β-actin is shown in the graph on the right. Panel B shows single-label immunofluorescence for MxA. Scale bar = 10 μm. Panel C shows double-label immunofluorescence for MxA and RTN4 of the periphery of cell in an IFN-treated culture (1000 IU/ml). The boxed inset in the merged panel is illustrated at higher magnification in the lower right. Scale bars = 10 μm. In Panel C, Pearson’s R (with Costes’ automatic thresholding) was 0.143 comparing RTN4 and MxA images.

**Fig 2:**
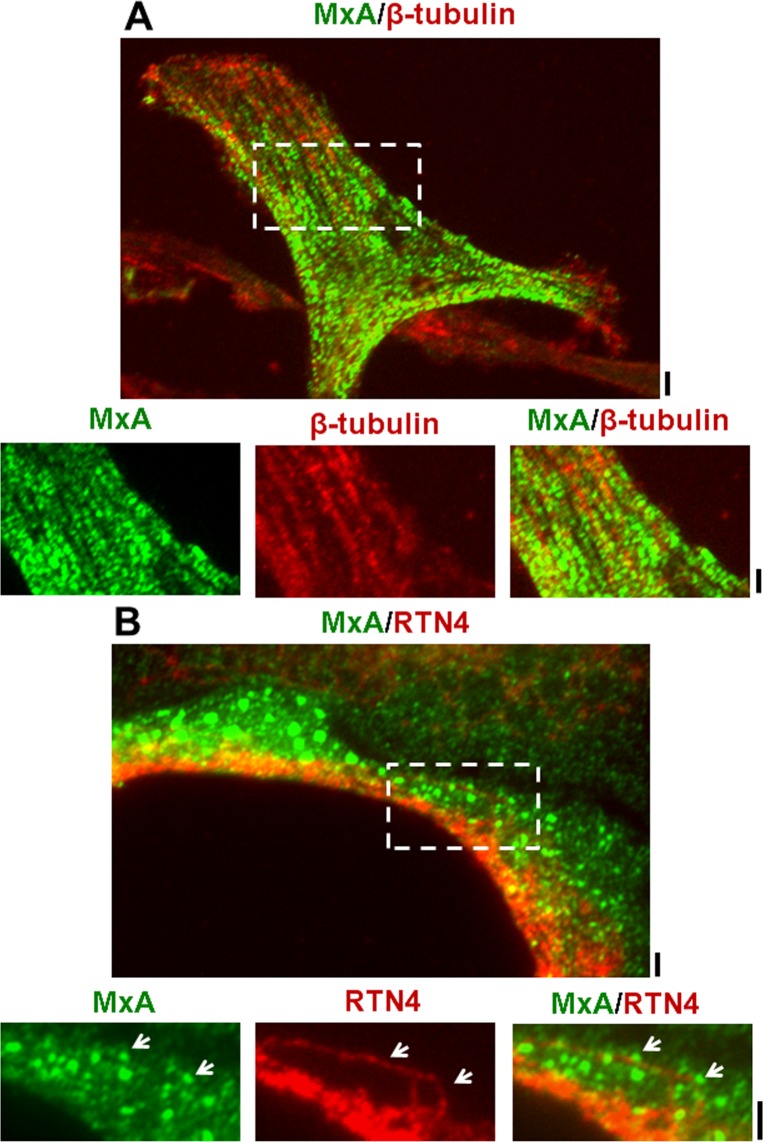
MxA-positive endosomes lie alongside microtubules and the RTN4-positive endoplasmic reticulum in HPAECs. Double-label immunofluorescence imaging was carried out on MxA-vector transfected HPAECs for MxA and RTN4 using a 40x water-immersion (Panel A) or 100x oil-immersion objective (Panel B). Panel A: Double-label analysis for MxA and β-tubulin (scale bar = 10 μm). The area in the white rectangle is also shown at higher magnification (scale bar = 5 μm).Panel B: Double-label analysis for MxA and RTN4 (a marker of the standard ER) (scale bar = 10 μm). The area in the white rectangle is also shown at higher magnification (scale bar = 5 μm). In Panel A, Pearson’s R (with Costes’ automatic thresholding) was 0.142 comparing tubulin and MxA images. In Panel B, Pearson’s R (with Costes’ automatic thresholding) was 0.23 comparing RTN4 and MxA images.

In contrast to prior reports that inferred that MxA colocalized with subcompartments of the endoplasmic reticulum (ER) [14,15,25,26,27,28], we found that in HPAECs MxA associated with endosomes which were distinct from ER tubules, and which lay alongside microtubules and ER tubules (Figs 1 and 2). We first investigated endogenously expressed MxA in primary HPAECs induced with IFN-α. Fig 1A illustrates a Western blot analysis showing that unstimulated HPAECs expressed very little MxA, and that IFN-α treatment markedly enhanced MxA expression. The single-label immunofluorescence analyses shown in Fig 1B show that this MxA was largely cytoplasmic. A double-label immunofluorescence analysis to evaluate the association of MxA with the standard RTN4-based ER summarized in Fig 1C showed (a) that the endogenously-expressed cytoplasmic MxA associated with endosomes, (b) that the MxA endosomes were clearly distinct from the ER tubules, and, (c) a subset of the MxA endosomes were aligned with and lay alongside ER tubules (see high-magnification of the boxed inset in the merged panel in Fig 1C lower right).

Fig 2 summarizes characterization of exogenously expressed MxA in HPAECs resulting from transient transfection with a constitutive expression vector for HA-tagged MxA. Fig 2A shows the dramatic alignment of MxA-endosomes alongside microtubules, and Fig 2B the presence of variably-sized MxA endosomes at the cell periphery, some of which aligned alongside ER tubules.

### MxA associates with early endosomes in HEK293T cells

**Fig 3:**
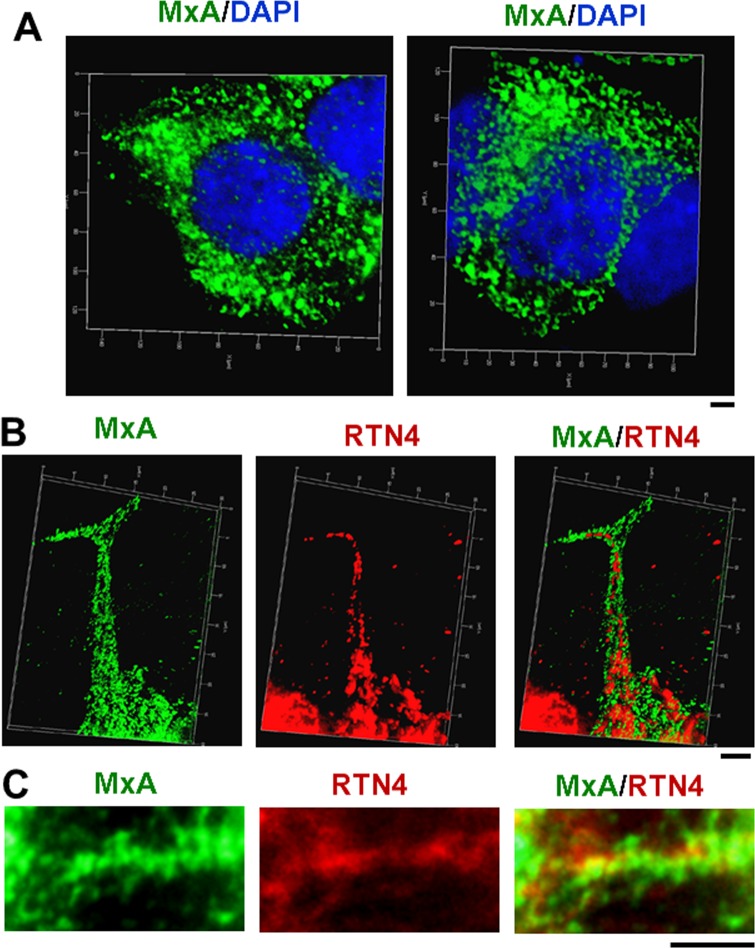
MxA-positive endosomes lie alongside the RTN4-positive endoplasmic reticulum in HEK293T cells. Single and double-label immunofluorescence imaging was carried out on MxA-vector transfected HEK293T cells using for MxA and for MxA and RTN4 using an 100x oil-immersion objective and z-stack image capture and deconvolution. Panel A: Images represent the combined deconvolved z-stack images of MxA of two individual MxA-overexpressing HEK 293T cells. Images show MxA-positive endosomes in the cytoplasm. Panel B illustrate merged MxA and RTN4 deconvolved z-stack images showing MxA-endosomes alongside RTN4-positive endoplasmic reticulum (but distinct from the ER). Panel C shows MxA-endosomes alongside RTN4-endosplasmic reticulum in from a second cell. Scale bars = 10 μm on Panel A, and 5 μm each in Panels B and C. In Panel B, Pearson’s R (with Costes’ automatic thresholding) was 0.064 comparing RTN4 and MxA images.

**Fig 4:**
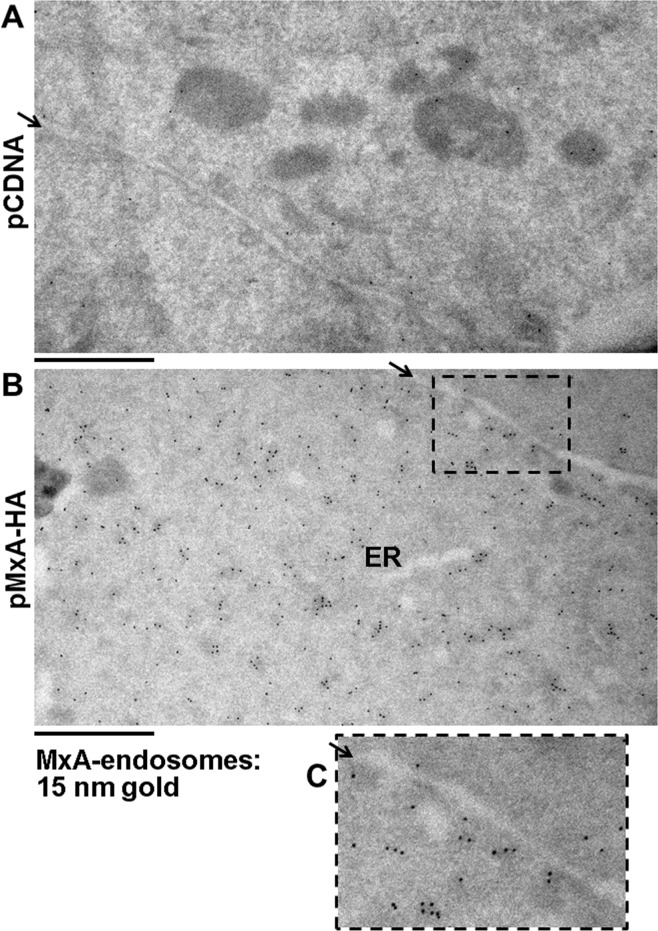
Evidence for MxA endosomes by immunoelectron microscopy. HEK293T cells were transfected with either pcDNA (Panel A) or the pMxA-HA vector (Panel B). One day later the respective cultures were processed for immuno-EM using anti-MxA rabbit pAb and Protein A-15 nm colloidal gold. Arrows point to the intercellular space between adjacent cells. The boxed inset in Panel B is shown at higher magnification in panel C. Scale bars = 1 μm.

Because of the well-known difficulty with carrying out replicable transient transfections of primary HPAEC cultures with significant frequency of transfected cells expressing an exogenous protein, we investigated exogenously expressed MxA in the more readily transfected HEK293T cells as has been used earlier in the MxA field [28,29]. An additional advantage of 293T cells has included a low-level of endogenous MxA expression (if at all) even after IFN-α treatment. Thus, these cells have been used by prior investigators in structure-function studies of mutants of MxA [28,29]. Fig 3A shows that exogenously expressed MxA in HEK293T cells was largely cytoplasmic and associated with endosomes. Fig 3B and 3C show that the MxA endosomes were distinct from RTN4-positive ER tubules, however, Fig 3C again shows that some of the MxA endosomes lay alongside ER tubules. The immuno-EM data summarized in Fig 4 confirm the association of MxA with endosomes both at the level of the plasma membrane vicinity and throughout the cytoplasm. The high-magnification inset (Fig 4C) of the boxed region in Fig 4B shows the association of MxA with endosomes at the level of the plasma membrane vicinity in MxA-expressing cells.

**Fig 5:**
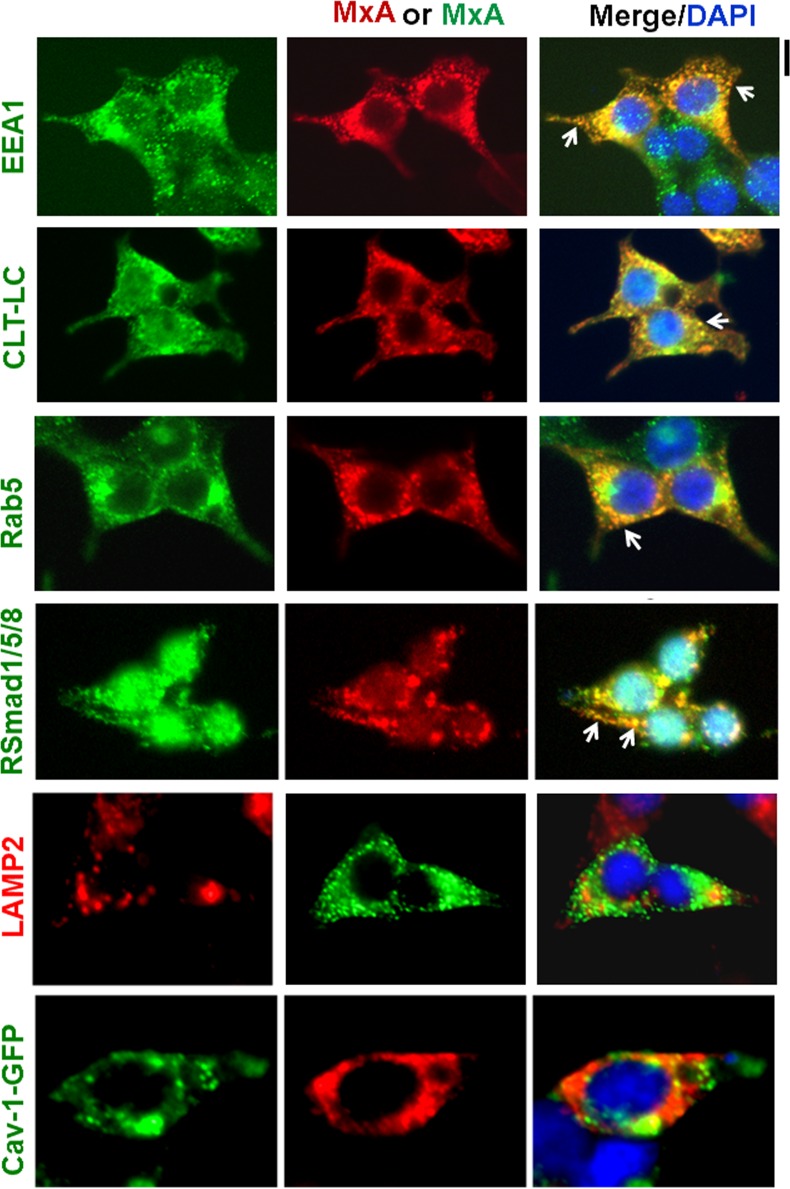
MxA endosomes carry early endosome markers and RSmad1/5/8. Double-label immunofluorescence imaging was carried out on MxA-vector transfected HEK293T cells for the indicated protein markers of early endosomes (EEA1, CLT-LC, Rab5), the transcription factors RSmad1/5/8 and caveolin-1-GFP and LAMP2 as indicated in Materials and Methods. Arrows indicate MxA-positive structures also positive for the respective second antigen. Scale bar = 10 μm. Pearson’s R (with Costes’ automatic thresholding) comparing MxA images with those of EEA1, CLT-LC, Rab5, RSmad1/5/8, LAMP2 and Cav-1-GFP were respectively 0.879, 0.704, 0.908, 0.709, 0.277 and 0.211.

A characterization of MxA endosomes in 293T cells using double-label immunofluorescence analyses (Fig 5) showed that these were positive for early endosome markers [early endosome antigen 1 (EEA1), clathrin-light chain (CLT-LC) and Rab 5] and also for the transcription mediators RSmad1/5/8. In contrast these endosomes were negative for caveolin-1 and lysosome membrane protein 2 (LAMP2). Thus MxA endosomes included protein markers associated with early endosomes in the clathrin-mediated endocytosis pathway.

### Endosome-associated BMP transcriptional signaling

**Fig 6:**
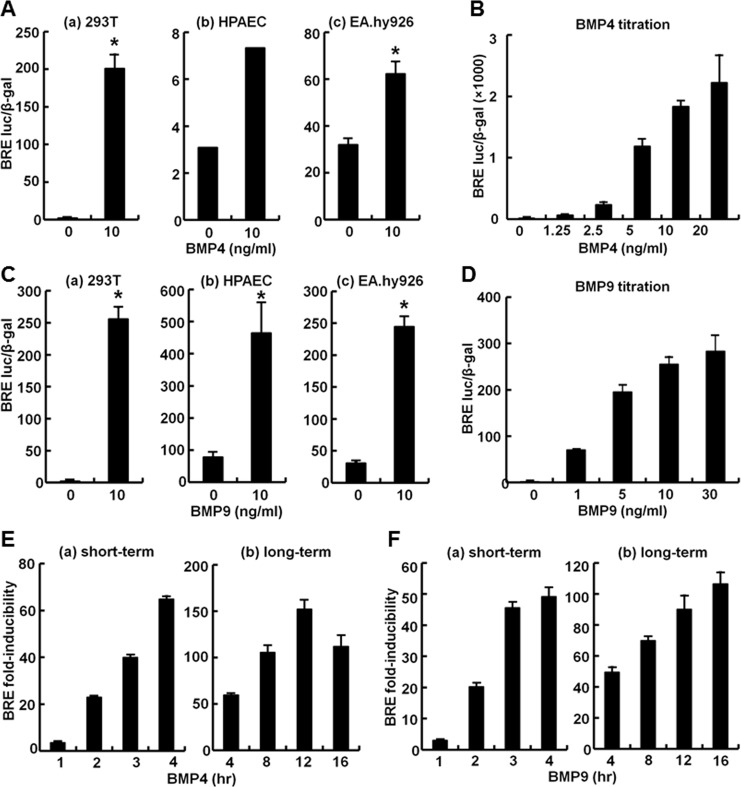
Response of pBRE-Luc in different cells and to different BMP concentrations and duration of BMP exposure. Cultures of human kidney cell line HEK293T (Panels A-a, C-a, B, D, E and F], human pulmonary arterial endothelial cells HPAEC (Panels A-b and C-b) or human umbilical vein endothelial cells EA.hy926 (Panels A-c and C-c) were transiently transfected with reporter construct pBRE-Luc together with constitutive β-galactosidase expression construct pCH110. 24 hr later the cultures were serum starved for 4 hr, followed by treatment with BMP4 or BMP9 for 15 hr at the indicated concentrations (Panels A-D) or 10ng/ml for the indicated times (Panels E and F). Cell lysates were assayed for β-galactosidase and luciferase activities. Within each experiment, the luciferase data were normalized for β-galactosidase activity in each extract. Each variable was investigated in single (A-b) or triplicate cultures (all other panels). Data are shown as mean ± SE. Asterisks indicate *p*<0.05 for the particular group when compared to the respective BMP-free groups.

In order to evaluate the functional contribution of these MxA endosomes on transcriptional signaling by BMP4/9 we first optimized a BMP-responsive-luciferase reporter construct (BRE-luc) known to respond to pSmad1/5/8 [30] for studies in endothelial cells and in 293T cells. Representative experiments are illustrated in Fig 6A and 6C. Primary HPAECs and the endothelial cell line EA.hy926, while responsive to BMP4 and BMP9, proved difficult to transfect replicably. In contrast the 293T cells showed a robust response to BMP4 and BMP9 (100-200-fold). In light of the practical difficulties encountered in working with primary endothelial cells (or even primary pulmonary arterial smooth muscle cells; not shown) in these transient luciferase-reporter transfection experiments, and the observation that MxA endosomes were also seen in 293T cells (Figs 3–5), we focused our effort on 293T cells in order to answer the question whether MxA endosomes affected BMP4/9 signaling. In 293T cells, both BMP4 and BMP9 showed transcription enhancing activity in the concentration range 1–2 ng/ml (Fig 6B and 6D). Clear transcriptional stimulation was evident within 2 hr of the beginning of ligand exposure and persisted for 12–16 hr (Fig 6E and 6F).

**Fig 7:**
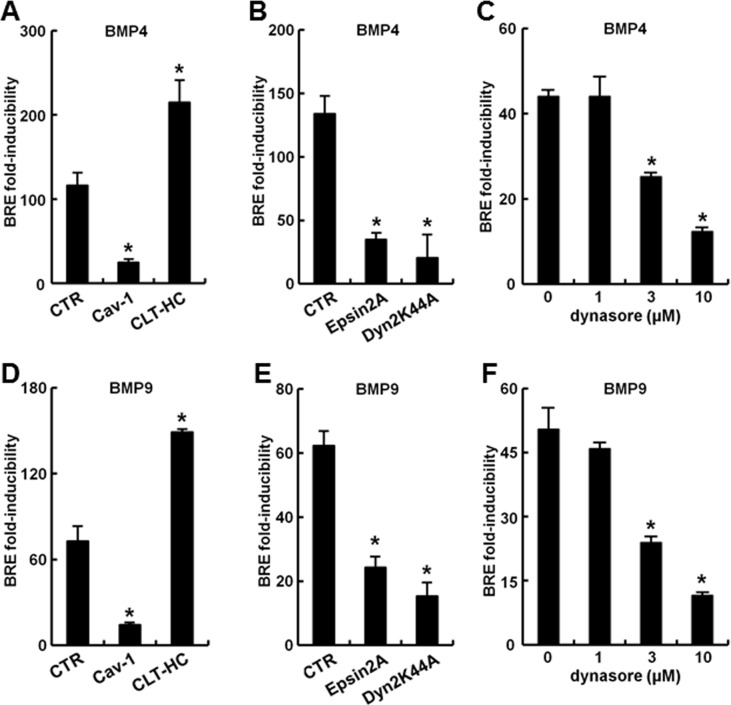
Effect of modifying the caveolin- or clathrin-mediated endocytic pathways on productive transcription following of BMP4 or BMP9 signaling. HEK293T cultures were transiently transfected with reporter construct pBRE-Luc and constitutive β-galactosidase expression construct pCH110 (Panels C and F) or additionally with the indicated expression constructs (Panels A, B, D and E). 24 hr later the cultures were serum starved for 4 hr, followed by treatment BMP4 or BMP9 at 10 ng/ml for 15 hr as indicated (Panels A, B, D and E) or with dynasore at the indicated concentrations together with BMP4 or BMP9 for 3 hr (Panels C and F). Cell lysates were assayed for β-galactosidase and luciferase activities. Within each experiment, the luciferase data were normalized for β-galactosidase activity in each extract. Each variable was investigated in triplicate. Data are shown as the mean ratio of the normalized luciferase activities in response to 10 ng/ml BMP4/9 compared to that in the absence of BMPs (“fold-inducibility”); error bars denote ± SE. Asterisks indicate *p*<0.05 for the particular group when compared to the control groups in Panels A, B, D and E or to the dynasore-free groups in Panels C and F. Cav-1, caveolin-1; CLT-HC, clathrin heavy chain.

The contribution of membrane-associated endocytic pathways in mediating BMP4 and BMP9-activated transcriptional signaling in the 293T cell-pBRE-luc reporter system used by us was investigated using two different strategies: by evaluating the effects on signaling of cotransfecting vectors expressing various endocytosis-mediator proteins, and by evaluating effects of the endocytosis inhibitor dynasore. The data in Fig 7A and 7D show that this signaling was stimulated by overexpression of the clathrin heavy chain (CLT-HC), but markedly inhibited by expression of wild-type (wt) caveolin-1 (cav-1). This inhibition by cav-1 expression was evident on both basal and BMP-inducible signaling even when a low concentration of the expression construct was used in the transfection reaction (in the range from 0.03 ng to 0.3 ng per culture; not shown). Parenthetically, 293T cells do not express detectable endogenous cav-1 as assayed by Western blotting of cell extracts, and thus the exogenously expressed cav-1 is the only pool of cav-1 present in these cells. The data summarized in Fig 7B and 7E show that overexpression of the dominant negative (DN) forms of epsin 2a and dynamin 2 (the K44A mutant) inhibited BMP4 and BMP9 signaling. Additionally, Fig 7C and 7F show that the endocytosis inhibitor dynasore reduced transcriptional signaling elicited by either BMP4 or BMP9. Taken together these data are evidence of the involvement of a clathrin-mediated endocytosis pathway in productive transcriptional signaling by BMP4 and BMP9 in the 293T cell-pBRE-luc system used by us, and a negative effect on signaling by cav-1. This dichotomy confirms previous observations about the regulation of TGF-β/Smad transcriptional signaling [18].

Thus, in light of (a) the strong BMP-stimulated inducibility of the pBRE-luc in 293T cells (Fig 6), and (b) confirmation of the involvement of membrane-associated trafficking in productive BMP4/9 signaling in these cells (Fig 7), we selected this cell system for experiments to investigate whether there might be any cross-talk between exogenously expressed MxA and transcriptionally productive BMP signaling.

### IFN-α and MxA enhance basal and BMP4/9-inducible transcriptional signaling

**Fig 8:**
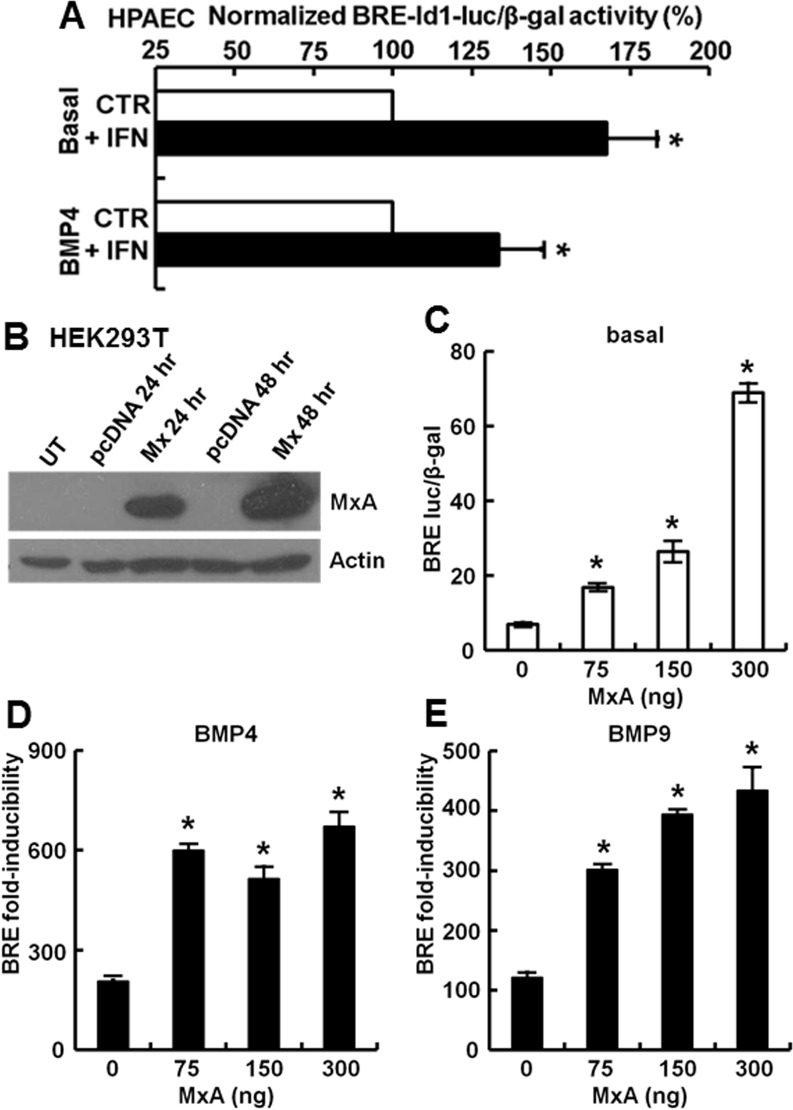
IFN-α and MxA enhance basal and productive transcription in response to BMPs. Panel A: HAPECs in 35 mm plates were transfected with the pBRE-luc reporter and the β-gal expression plasmids using Lipofectamine 3000. The next day the cultures were either left untreated or exposed to IFN-α (3000 IU/ml) for 12 hrs. Cultures were then either continued as such (“Basal”) or exposed to BMP4 (30 ng/ml) for another 15 hr. Graph summarizes pooled data from two independent experiments in terms of the BRE-Id1-luciferase/β-gal activity normalized to the IFN-free groups (“CTR”) as 100%. Asterisks indicate *p*<0.05 comparing the IFN-containing groups with the IFN-free groups. Panel B: Expression of MxA in HEK293T cultures transfected with the MxA-HA expression vector. Whole-cell lysates were prepared 24 or 48 hr after transient transfection. Immunoblots were carried out using equal aliquots protein (80 μg) from such extracts. Panels C-E: HEK293T cultures were transiently transfected with reporter construct pBRE-Luc together with the constitutive β-glycosidase expression construct pCH110 and indicated amounts of the constitutive MxA-HA expression construct. Twenty-four hr later the cultures were serum starved for 4 hr, followed by exposure to BMP4 or BMP9 at 10 ng/ml for 15 hr (Panels D and E) or left untreated (Panel C) in triplicate for each variable. Cell lysates were assayed for β-galactosidase and luciferase activities. Panel C shows the increase in basal luciferase activity in response to MxA alone; Panels D and E show data for fold-inducibility (mean ± SE). Asterisks indicate *p*<0.05 for the particular group when compared to the respective MxA-free groups in Panel C, D and E.

The question of whether IFN-α *per se* modulated Smad-dependent transcriptional signaling was addressed in primary cultures of HPAECs under conditions that showed a marked induction of endogenous endosome-associated comparable to those in Figs 1 and 2. Fig 8A summarizes pooled data from two independent experiments in which HPAEC cultures were transfected with the BRE-luc reporter using Lipofectamine 3000. There was an increase in the BRE-Id1-luc reporter activity in cultures treated with IFN-α whether by itself or together with BMP4. However, similar to the data summarized in Fig 6A, the magnitude of the BRE-luc activity in HPAECs was lower than that which can be observed in 293T cells. However, in 293T cells which give a far greater BMP-inducible luciferase signal (Fig 6A), IFN-α induced MxA only minimally, if at all ([29], and data not shown). Thus as a compromise, we investigated whether expression of MxA in 293T cells by itself using an exogenous expression vector construct had a concentration-dependent enhancing effect on basal transcription of the BRE-luc reporter (Fig 8B). The data in Fig 8C show that expression of exogenous MxA in 293T cells enhanced basal BRE-Id1-luciferase activity. Remarkably, exposure to BMP4 or BMP9 in the presence of MxA caused an even further increase in levels of fold-inducibility of BRE-luc transcription (Fig 8D and 8E). We therefore used 293T cells for further mechanistic dissection of the effects of MxA on BMP/Smad transcriptional signaling.

### Enhancement by MxA of BMP4/9 signaling involves endocytosis and microtubules

**Fig 9:**
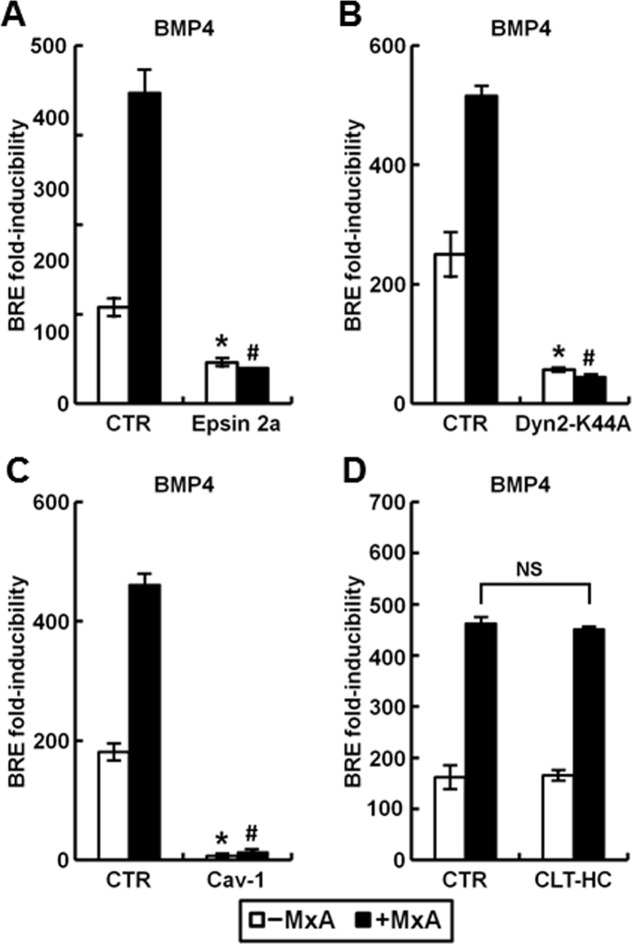
Perturbation of the clathrin-mediated endocytic pathway and increased caveolin expression abrogated the enhancing effect of MxA on transcriptional activation by BMP4. Panels A-D: HEK293T cultures were transiently transfected with reporter construct pBRE-Luc together with constitutive β-galactosidase expression construct pCH110 with (solid columns) or without MxA (open columns) and the indicated expression constructs. Twenty-four hr later the cultures were serum starved for 4 hr, followed by treatment with BMP4 at 10 ng/ml for 15 hr or left untreated. Data are shown as fold-inducibility (mean ± SE). Asterisk and pound signs indicate *p*<0.05 for the indicated group when compared to the–MxA control groups or +MxA control groups, respectively. “NS” denotes for *p*>0.05 for the indicated groups.

**Fig 10:**
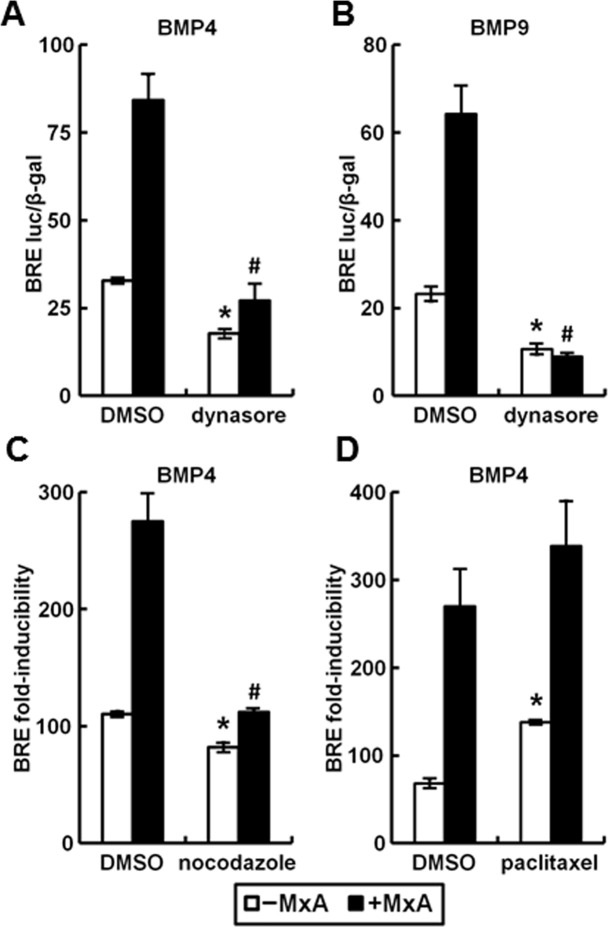
Perturbation of the clathrin-mediated endocytic pathway and disruption of microtubules dynamics abrogated the enhancing effect of MxA on transcriptional activation by BMP4. HEK293T cultures were transiently transfected with reporter construct pBRE-Luc together with constitutive β-galactosidase expression construct pCH110 with (solid columns) or without MxA (open columns). 24 hr later the cultures were serum starved for 4 hr and then pretreated with 15 μM dynasore (Panels A and B), 10 μg/ml nocodazole (Panel C) or 20 nM paclitaxel (Panel D) at 37°C for 30 min, followed by treatment with 0 or 10 ng/ml BMP4 or BMP9 together with each chemical at 37°C for another 3 hr (Panels A and B) or 15 hr (Panels C and D). Cell lysates were assayed for β-galactosidase and luciferase activities. Within each experiment, the luciferase data were normalized for β-galactosidase activity in each extract. Each variable was investigated in triplicate. Data are shown as mean ± SE; as normalized luciferase activity in Panels A and B, and as fold-inducibility in Panels C and D. Asterisk and pound signs indicate *p*<0.05 for the indicated group when compared to the–MxA DMSO group or the +MxA DMSO groups, respectively.

The involvement of endocytosis in the enhancement by MxA of BMP signaling was investigated using expression vectors of endocytosis regulatory proteins and the endocytosis inhibitor dynasore. The data in Fig 9A, 9B and 9C show that the enhancing effect of MxA on BMP4-induced transcriptional signaling was inhibited by overexpression of the DN mutants of epsin2a and dynamin 2, and by wt cav-1. In contrast increased expression of clathrin-HC did not affect the MxA-produced enhanced signaling (Fig 9D). Furthermore, the data in Fig 10A and 10B show that dynasore inhibited the enhancement of BMP4- and BMP9-induced signaling by MxA. Moreover, Fig 10C shows that nocodazole, a microtubule inhibitor, reduced the effect of MxA. In contrast, paclitaxel, a microtubule stabilizer, did not interfere with the enhancement of inducible transcription by MxA (Fig 10D). These data indicate that the enhancement of BMP-inducible signaling by MxA involved the clathrin-endocytic pathway and microtubules.

**Fig 11:**
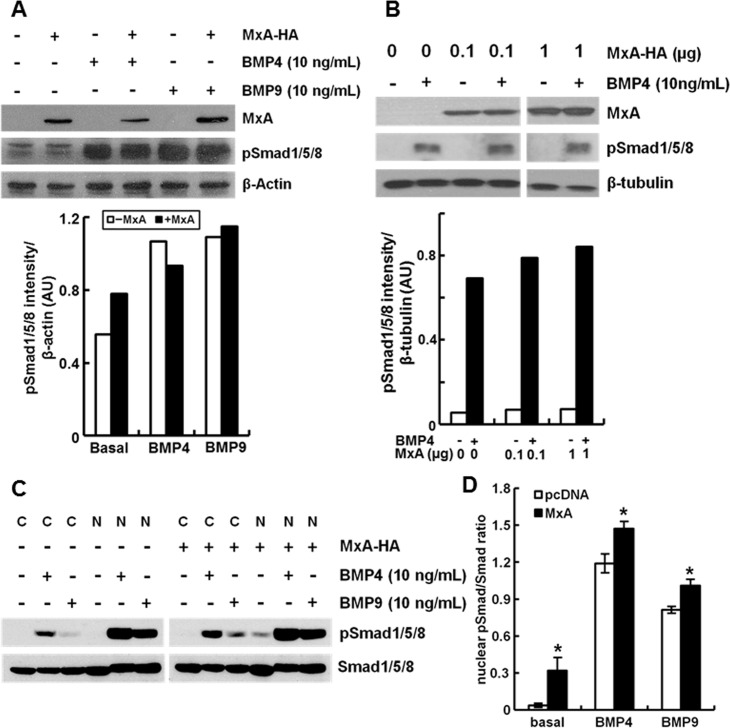
MxA expression does not affect whole-cell phospho-RSmad content but enhances the nuclear pRSmad/RSmad ratio in cells exposed to BMP4 or BMP9. HEK293T cultures were transfected with pcDNA or MxA expression constructs (0.6 μg in Panels A and C, and the indicated amounts in B). After 24 hr, cell cultures were left untreated or exposed to BMP4 or BMP9 at 10 ng/ml for 15 hr. Whole-cell extracts were prepared in the experiments in Pansls A and B, while the cells were fractionated into a cytoplasmic and nuclear extract in Panel C using the hypotonic swelling method. Immunoblotting assays were carried out using protein-matched aliquots (80 μg/lane in Panels A and B, and 50 μg/lane in Panel C) of the respective extracts. Blots were probed for MxA and phospho-RSmad as indicated; β-actin and β-tubulin were used as internal loading control. Quantitation of the blots shown in Panels A and B in terms of pRSmad activation normalized to β-actin (for Panel A) or β-tubulin (for Panel B) is shown in the graphs below each respective blot. Panel D: Data illustrate the ratio of pRSmad/RSmad in the respective nuclear fractions (mean ± SE) calculated from experiment as in Panel C. Asterisks indicate *p*<0.05 for the particular group compared to respective MxA-free group.

The increase in BMP-induced transcriptional output in MxA expressing cells (Fig 8C and 8D) was evident without commensurate BMP-induced increase in *whole* cell BMP-stimulated pSmad1/5/8 levels (Fig 11A and 11B). However, following cell fractionation, an increase in the ratio of nuclear pSmad1/5/8 to unphosphorylated Smad was evident even in these transient transfection experiments (Fig 11C and 11D), suggesting a more efficient transit to the nucleus in the presence of MxA.

### Can MxA rescue the inhibition of transcriptional signaling by the F14, KDF and R899X mutants of BMPR2?

**Fig 12:**
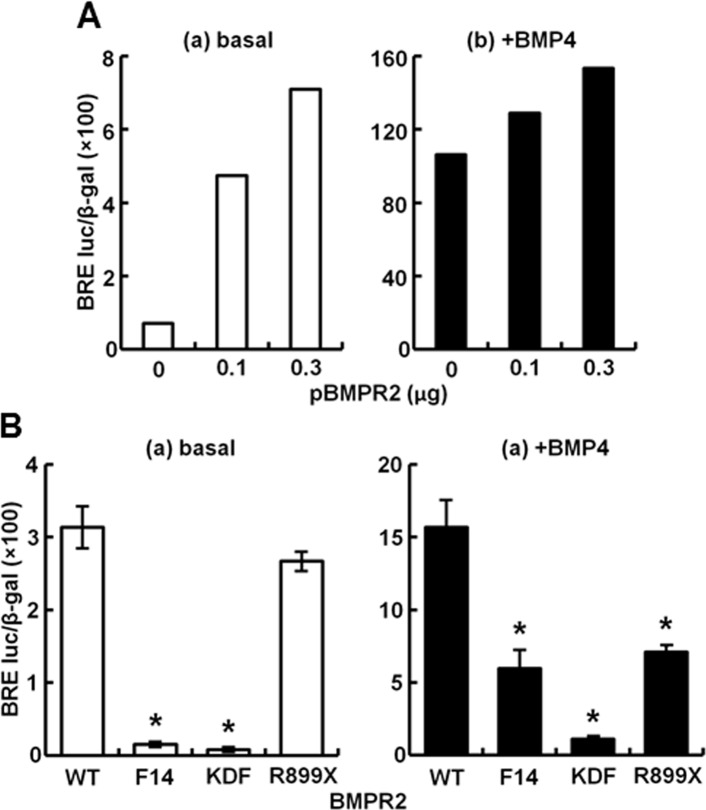
Effect of expression of wild-type (WT) and mutated BMPR2 species on productive transcription in response to BMP4. HEK293T cultures were transiently transfected with reporter construct pBRE-Luc together with constitutive β-galactosidase expression construct pCH110 and the indicated type and amounts of WT (Panels A and B) or mutated (Panels B) BMPR2 expression constructs. Tweinty-four hr later the cultures were serum starved for 4 hr, followed by treatment with BMP4 at 10 ng/ml (+BMP4) for 15 hr. Cell lysates from uninduced (“basal”) or induced (“+BMP4”) cultures were assayed for β-galactosidase and luciferase activities; each variable was evaluated in triplicate in panel B. Within each experiment, the luciferase data were normalized for β-galactosidase activity in each extract. Data are shown as mean ± SE. Asterisks indicate *p*<0.05 for the particular group when compared to the WT group.

The F14, KDF and R899X mutants of BMPR2 correspond to hereditary PAH-disease associated autosomally-dominant mutants which are known to mislocalize in vascular cells and to inhibit BMP-responsive signaling in 293T cells [1,2,3,31](details of the mutations and the subcellular localization are in Materials and Methods, and in ref. [31]). We first confirmed that in our hands in 293T cells that both basal and BMP-induced luciferase reporter activities were enhanced by additional transfection with a wt BMPR2 construct (Fig 12A). Next we verified that expression constructs corresponding to the F14, KDF and R899X mutants inhibited basal and BMP4-induced signaling (Fig 12B). We then evaluated whether MxA could overcome this inhibition.

**Fig 13:**
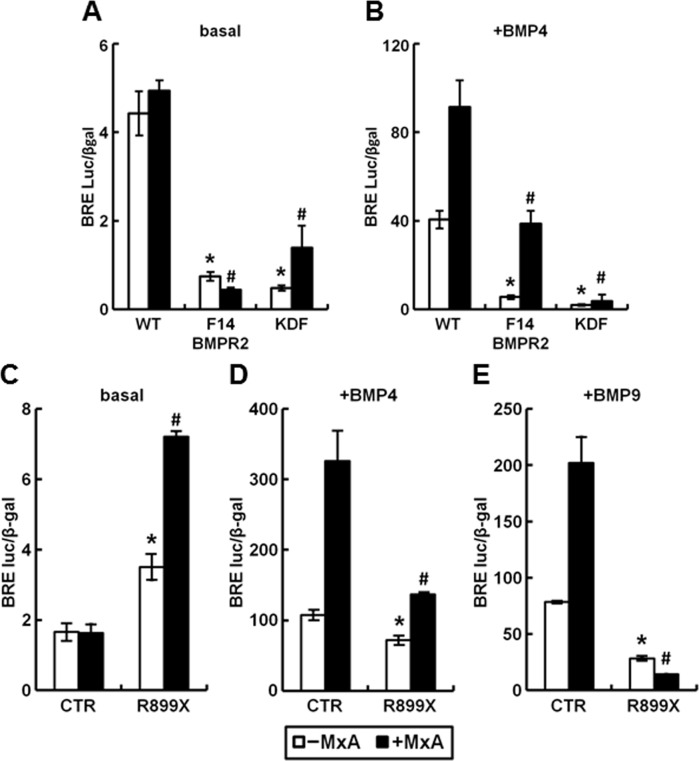
MxA partially rescues the inhibition of BMP4 signaling PAH-disease associated mutants of BMPR2 (F14, KDF and R899X). HEK293T cultures were transiently transfected with reporter construct pBRE-Luc together with constitutive β-galactosidase expression construct pCH110 with (solid columns) or without MxA (open columns) and the indicated BMPR2 expression constructs (WT, F14, KDF and R899X). Twenty-four hr later the cultures were serum starved for 4 hr followed by treatment with 0 (Panels A, C; “basal”) or 10 ng/ml BMP4 (Panels B, D) or BMP9 (Panel E) for 15 hr. Cell lysates were assayed for β-galactosidase and luciferase activities. Within each experiment, the luciferase data were normalized for β-galactosidase activity in each extract. Each variable was investigated in triplicate. Data are shown as mean ± SE. Asterisks and pound signs indicate *p*<0.05 for the indicated group when compared to the–MxA control groups or +MxA control groups, respectively.

The data in Fig 13A and 13B show again that MxA was able to enhance signaling in the presence of wt BMPR2. Moreover, MxA also further enhanced BMP4 and BMP9 signaling in control cells with endogenous BMPR2 alone (CTR groups in Fig 13D and 13E). Remarkably, MxA enhanced basal signaling in the presence of the KDF and R899X mutants (Fig 13A and 13C), and partially rescued BMP4-induced signaling in the presence of the F14 and R899X mutants (Fig 13B and 13D). Taken together, these data point to the ability of MxA to at least partially rescue BMP signaling in the presence of some of the autosomally-dominant PAH-disease associated mutants of BMPR2.

## Discussion

**Fig 14:**
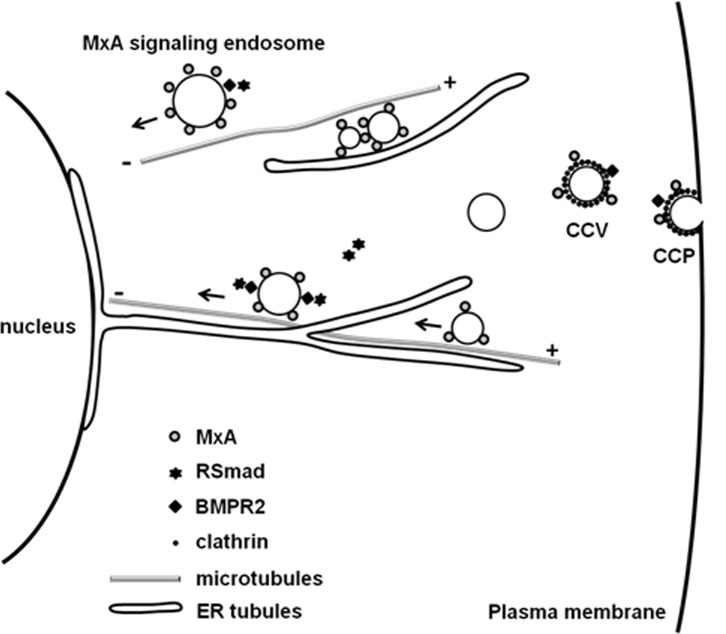
MxA-signaling endosome model. Schematic shows the proposed scaffolding interactions between MxA-endosomes, microtubules and ER that likely contribute to vectorial BMP signal transit through the cytoplasm. CCP, clathrin coated pit; CCV, clathrin-coated vesicle.

The present studies identify a previously unrecognized cellular function of MxA. This dynamin-family GTPase stimulates productive transcription activated by BMP4 and BMP9. We determined that productive signaling initiated by both BMP4 and BMP9 transits along the clathrin-mediated endocytosis pathway in association with microtubules. Immunofluoresence and immuno-electron microscopy showed the association of MxA with early endosomes, and these, in turn, were often observed alongside microtubules [32,33] and ER tubules. The latter organelles likely serve a scaffolding role in vectorial endosome trafficking as recently proposed by Voeltz and colleagues [24]. In applying the Voeltz scaffolding model to the present data (Fig 14), we propose that MxA (dimeric or oligomeric) might serve as a tether helping target signaling endosomes to stay alongside microtubules and ER for purposes of vectorial transit towards the nucleus. Numerous prior studies have emphasized the role of this vectorial transit of “signaling endosomes” as contributing to the speed and efficiency of signal spread from the plasma membrane to the vicinity of the nucleus [17,18,19,34,35,36].

There have been several reports in the literature between 2002 and 2015 in which investigators inferred that MxA associated with subcompartments of the smooth ER [12,13,14,15,16,25,26,27,28]. Our data agree with the previously published images of variably-sized punctate MxA-positive structures in the cytoplasm, but our conclusions disagree with the previous inferences about ER localization. We note first of all that none of these prior studies dealt with the localization of IFN-α-induced endogenously expressed MxA (as in the present Fig 1). Second, even with respect to the localization of exogenously expressed MxA, we note that the claim of an ER localizationof MxA derived from these prior studies is suspect in that the prior investigators used syntaxin 17 (STX17) or autocrine motility factor receptor (AMF-R) as purported markers of the ER, and not a structural protein of the smooth ER such as reticulon-4 in their co-localization studies. Neither of STX17 nor AMF-R are considered markers of the ER; STX17 is a marker of autophagosomes and shuttles among other cytoplasmic organelles [37,38], and AMF-R shuttles between the plasma membrane and intracellular organelles [15]. In contrast to these prior studies, we used the ER structural protein RTN4 as a marker in double-label co-localization studies and show that MxA endosomes, although they lie alongside ER tubules, are distinct from the standard RTN4-based ER. Moreover, others have also pointed to an endosomal association of MxA [17,39,40], as well as the presence of MxA in cross-immunoprecipitable complexes with dynamin and amphiphysin II [39].

In the present studies of BMP4 and BMP9 signal transduction, we observed that disruption of normal endocytosis resulted in inhibition of the transcriptional function of BMP signaling. In these studies we used different genetic and chemical approaches to inhibit endocytosis, to demonstrate the requirement for endocytosis in the transcriptional outcome of BMP4 and BMP9 signaling. As with other cytokines, we observed that clathrin-mediated endocytosis was involved in productive transcriptional signaling, while caveolin-associated endocytosis was inhibitory [18]. MxA was able to further stimulate signaling along the clathrin pathway but unable to overcome the inhibition by caveolin-1. These data are in accord with observations of a positive transcriptional outcome through signaling along the clathrin-endosome pathway but a negative outcome by caveolin-1-endocytosis (the inhibitory effects of the caveolin pathway has been previously observed) [18,34,35,36]. The mechanisms that determine the sorting balance between the clathrin- and caveolin endocytic pathways and their extent of cross-talk under different physiological conditions (e.g. cell stress, nutrient starvation, and hypoxia) remain to be explored.

A subset of MxA-endosomes were located alongside ER tubules in both 293T cells and endothelial cells. This spatial localization pattern of endosomes along the ER was similar to that reported by the Voeltz group [22,24], implying the coupling between signaling endosomes and ER tubules. We note that it has been already reported that MxA can bind microtubules and the ER [26,32,33]. We suggest that MxA serves as a tether enabling signaling endosomes (which are MxA-positive) to bind to microtubules and ER tubules as scaffolds during their transit towards nucleus (Fig 14). Such a model would provide rapid and vectorially directed transport from the plasma membrane to the nuclear pore region. As a member of dynamin GTPase superfamily, MxA shares a highly conserved GTPase domain with dynamins which are involved in the piching off of endocytic vesicles from the plasma membrane. Therefore, an alternative mechanism for the enhancing effect of MxA on BMP4/9 signaling may include enhancing the efficiency of membrane fission at the clathrin-coated pits to facilitate a more rapid release of signaling endosomes into the cytoplasm contributing to enhanced BMP signaling (Fig 14).

The initial impetus to investigate the effect of MxA on BMP4 and BMP9 signaling were the observations by George *et al* [10] that IFN-α contributed to hypoxic PAH in a mouse model, and the converse observations by Bauer *et al* [11] that IFN-α was protective. In our experiments in HPAECs we observed that IFN-α enhanced basal and BMP4-stimulated BRE-Id1-luc transcriptional activity, an observation consistent with a protective effect of IFN-α in PAH. Moreover, the present observations that MxA expression enhanced signaling by BMP4 and BMP9 are also aligned with a protective role of IFN-α. The IFN-α-induced protein MxA/Mx1 enhanced the vascular quiescence signaling of BMP4/9. These observations are also consistent with the ability of MxA to partially rescue the inhibition of BMP signaling by PAH-disease-associated mutants of BMPR2 with different effects on different mutants. However, MxA is only one of many hundreds of genes that are upregulated by IFN-α, and thus the observations that a small subset of patients given IFN-α develop PAH may reflect the actions of these myriad of other changes in IFN-treated vascular tissues in addition to upregulation of MxA.

To summarize, we demonstrate the association of MxA with early endosomes in HPAECs and in 293T cells. These MxA endosomes stimulate the directionally vectorial transit of the transcription activation signal generated by BMP4 and BMP9 at the plasma membrane along the clathrin-mediated endocytic pathway. The present studies represent the first investigation of cross-talk between MxA and BMP4/9 at the level of intracellular signal transit. Overall, the data identify MxA as a novel candidate mechanism to consider by which Type I IFNs could influence the pathogenesis of PAH and other vascular diseases.

## Materials and Methods

### Cells and cell culture

Human kidney cancer cell line HEK293T was obtained from the American Type Culture Collection (ATCC). These cells were grown in DMEM supplemented with 10% v/v fetal bovine serum (FBS). HEK293T cells were seeded into 6-well, 24-well plates or 35mm-dishes coated with fibronectin, collagen and bovine serum albumin (respectively 1 μg/ml, 30 μg/ml and 10 μg/ml in coating medium) [41,42,43]. Primary human pulmonary arterial endothelial cells (HPAEC) were purchased from Clonetics (San Diego, CA). These were seeded into T-25, T-75 or 6-well plates coated with fibronectin, collagen and bovine serum albumin (respectively 1 μg/ml, 30 μg/ml and 10 μg/ml in coating medium) [41,42,43]. HPAECs were grown in Medium 200 supplemented with low serum growth supplement LSGS (Cascade Biologics, Carlsbad, CA) and were used between passages 4 and 10 [44]. The human endothelial cell line EA.hy926 was a gift from Dr. Yang-Ming Yang (Dept. Cell Biology & Anatomy, New York Medical College) and grown in DMEM supplemented with 10% v/v fetal bovine serum and 100 μM hypoxanthine, 0.4 μM aminopterin, and 16 μM thymidine [31]. Phase contrast microscopy was carried out daily and at the conclusion of each experiment using a Nikon Diaphot Microscope and a Nikon Coolpix digital camera.

### Growth factors, cytokines, and treatment

Recombinant human BMP4 (#314-BP) and BMP9 (#3209-BP) were purchased from R&D System (Minneapolis, MN). Recombinant human interferon-alpha 2a (IFN-α2a) was purchased from BioVision (Milpitas, CA). Cell cultures were grown to confluence at 37°C. For BMP treatment cultures were washed twice with phosphate-buffered saline, replenished with serum-free medium for 4–8 hr, and then exposed to the recombinant cytokine or growth factors indicated overnight (12–16 hr).

### Plasmids and transient transfection experiments

The HA-tagged MxA expression vector (cloned into a pcDNA3 vector) was a gift from Dr. Otto Haller (University of Freiburg, Germany). Caveolin-1 (GFP fused) expression vector was from Dr. Jeffrey Pessin (SUNY Stony Brook). Clathrin heavy chain expression vector (CLTC) (TC117104) (referred to as “CLT-HC” in this document) (cloned to pCMV6-XL5 vector) was purchased from OriGene (Rockville, MD). Clathrin light chain expression vector (referred to as “CLT-LC” or “CLC” in this document) was from Dr. Tomas Kirchhausen (Harvard Medical School). Dynamin2-K44A expression vector was from Dr. Lois Greene (NHLBI). Epsin 2a expression vector were from Dr. Richard Jove (University of South Florida). Constitutive expression vectors for β-galactosidase (pCH110) and for Flag-tagged BMPR2 proteins [vectors were 3xC-terminal Flag tag/CMV in pCIneo corresponding to wild-type (wt); “F14”—exon 3 T354G (Cys118Trp), “KDF”—exon 8 C944T (Arg322X), and the 1x Flag-tagged BMPR2-R899X protein were gifts from Dr. Yang-Ming Yang (Dept. of Cell Biology & Anatomy, New York Medical College [31]. Plasmid transfections were carried out using PolyFect (Qiagen, Valencia, CA) or Lipofectamine 3000 (for HPAECs; Invitrogen, Waltham, MA) and the respective manufacturer’s protocols.

### Construction of BMP reporter pBRE-Luc

The BMP reporter pBRE-Luc was constructed based upon the BMP-responsive Smad1/5/8 binding enhancer elements that regulate the human Id1 promoter [30]. Briefly, the synthetic oligonucleotides 5’- CCGC$GGCGC\underline{C}$$\underline{AGC}$CTGA$\underline{CAGC}$CCGTCCT_GGC_${}_{\underline{\underline{G}}}\underline{\underline{TCT}}$AACG$\underline{\underline{GTCT}}$GAC -3’ (sense) and 5’- TCGAGTC$\underline{\underline{AGAC}}$CGTT$\underline{\underline{AGA}}{}_{\underline{\underline{C}}}$_GCC_AGGACGG$\underline{GCTG}$TCAG$\underline{GCT}$$\underline{G}GCGCC$GCGGAGCT -3’ (antisense), containing two CAGC sites (underlined), one GGCGCC palindrome (highlight in grey), one GGCG site (subscript), two SBE (double underlined), SacI 5’-overlapping and XhoI 3’-overlapping on the sense strand and corresponding antisense strand, were ligated and inserted into SacI and XhoI sites in pGL4.48 [luc2P/SBE/Hygro] vector from Promega (Madison, WI) to substitute the original SBE fragment and generate the luciferase reporter construct with one copy of BRE fragment flanked by SacI and XhoI (BREx1Luc). To generate a BRE luciferase reporter with two BRE fragments tandemly linked, another copy of BRE fragment but franked by XhoI and BglII annealed from synthetic oligonucleotides 5’- TCGAGCGC$GGCGC\underline{C}$$\underline{AGC}$CTGA$\underline{CAGC}$CCGTCCT_GGC_${}_{\underline{\underline{G}}}\underline{\underline{TCT}}$AACG$\underline{\underline{GTCT}}$GAA -3’ and 5’- GATCTTC$\underline{\underline{AGAC}}$CGTT$\underline{\underline{AGA}}{}_{\underline{\underline{C}}}$_GCC_AGGACGG$\underline{GCTG}$TCAG$\underline{GCT}$$\underline{G}GCGCC$GCGC -3’ were inserted into BREx1Luc in XhoI and BglII sites. Thus we generated a BRE luciferase reporter construct with two copies of BRE fragments tandemly linked and flanked by SacI and BglII, called pBRE-Luc.

### BRE-Luciferase reporter assays

Cultures (in 6- or 24-well plates or 35 mm plates) were transfected with pBRE-Luc, pCH110 and other plasmids indicated in different experiments, followed by treatment with respective cytokines as described in individual experiments. Typically, 3 x 10^5^ cells were plated in a 35 mm dish or in a well of a 6-well plate, and the cultures used for transfection the next day. Alternatively, 1.5 x 10^5^ cells/well were plated in a 24-well plate and the cultures used for transfection the next day. Each variable was typically evaluated in triplicate; each particular experiment was independently replicated 2–4 times. At the conclusion of each experiment the cells were harvested, lysed and the expression of luciferase assayed using the Luciferase Assay System (E1500) purchased from Promega (Madison, WI) following the manufacturer’s instructions. The lysate were also used to assay for expression of β-galactosidase activity by using chlorophenolred-β-D-galactopyranoside (CPRG) (10 884 308 001) purchased from Roche Diagnostics (Indianapolis, IN) following a protocol provided by the manufacturer. Typically, luciferase activity is expressed as mean activity per variable (n = 3 each) ± SE after normalization with the β-galactosidase activity in the respective samples.

### Cell extracts and Western blotting

Whole cell extracts, as well as cytoplasmic and nuclear extracts using hypotonic swelling and Dounce breakage, were prepared from HEK293T cells as described previously [31,41,42] and citations therein). Western blotting was carried out using 8, 10 or 12% SDS-polyacrylamide gels under reducing denaturing conditions as per procedures and protocols provided by Cell Signaling Technology Inc. and ECL detection kit purchased from Thermo Scientific (34077) (Rockford, IL) and Michigan Diagnostics (FWPD02-25) (Royal Oak, MI). Multiple exposures of each blot were obtained to ensure that each of the signals was within the linear range. Western blot signals were quantitated using a Hoefer Scientific GS-300 scanning densitometer. Linear brightness and contrast adjustments of scanned images of blots were made using the NIH Image J software. Images in blots were quantitated for optical density also using the NIH Image J software.

### Immunofluorescence imaging

Cultures were fixed using cold paraformaldehyde (4%) for 1 hr and then permeabilized using a buffer containing digitonin (50 μg/ml)/sucrose (0.3M) [10,31,42,43] or 0.3% Triton X-100. Immunofluorescence assays were carried out using antibodies from specific sources and corresponding to the specific catalog numbers indicated in parentheses below (dilution range 1:100 to 1:1000) as described earlier [41,42]. Respective immunofluorescence was imaged as previously reported [42] using a Zeiss AxioImager M2 motorized microscopy system with Zeiss W N-Achroplan 40X/NA0.75 or Zeiss EC Plan-Neofluor 100X/NA1.3 oil objectives equipped with an high-resolution RGB HRc AxioCam camera and AxioVision 4.8.1 software in a 1388 x 1040 pixel high speed color capture mode. Images in z-stacks were captured using Zeiss AxioImager software; these stacks were then deconvolved and rendered in 3-D using the 64-bit version of the Zeiss AxioVision software. Deconvolution of 2-D images was carried out using Image J software. Controls included secondary antibodies alone, peptide competition assays and multiple different antibodies towards the same antigen. All data within each experiment were collected at identical imaging settings. Colocalization analyses of structures in regions of interest in double-label immunofluorescence images were carried out using Costes’ automatic thresholding and the Pearson’s R correlation metric using the colocalization plugin in NIH Image J software (R = 1.0 represents complete overlap, R = 0.0 represents no overlap; R ≥ 0.7 indicates strong colocalization).

### Electron microscopy

For immuno-EM, the cultures were fixed with paraformaldehyde (4%) for 20 min at 37°C, and then another 40 min at room temperature [17,41,43]. After fixation, the cells were washed with ice-cold phosphate-buffered saline (PBS), scraped into an Eppendorf tube and pelleted by centrifugation for 15 sec. The cell pellets were continued to be fixed in freshly made 2% paraformaldehyde in PBS containing 0.2% glutaraldehyde, pH 7.2–7.4 for 4 hours at 4°C. After washing with PBS, the cells were embedded with 10% gelatin, infused with sucrose, and cryosectioned at 80 nm thickness onto 200 mesh carbon-formvar coated copper grids. For single MxA labeling, the grids were blocked with 1% coldwater fish skin gelatin (Sigma) for 5 min, incubated with MxA antibody (rabbit pAb) in blocking solution for 2 hours at room temperature. Following washing with PBS, gold-conjugated secondary antibodies (15 nm Protein A- gold, Cell Microscopy Center, University Medical Center Utrecht, 35584 CX Utrecht, The Netherlands, or 18 nm Colloidal Gold-AffiniPure Goat Anti-Rabbit IgG, Jackson ImmunoResearch Laboratories, Inc., West Grove, PA) were applied for 1 hr. The grids were fixed in 1% glutaraldehyde for 5 min, washed with distilled water, contrasted and embedded in a mixture of 3% uranyl acetate and 2% methylcellulose in a ratio of 1 to 9. Stained grids were examined using a Philips CM-12 electron microscope (FEI; Eindhoven, The Netherlands) and images photographed with using a Gatan (4K x 2.7K) digital camera (Gatan, Inc., Pleasanton, CA).

### Antibody reagents

Mouse mAb to clathrin light chain (#12735), and to β-tubulin (#23949), rabbit pAbs to human MxA (also referred to as human Mx1)(H-285)(#50509), to Flag/OctA (D-8) (#807), and to Smad1/5/8 (N-18) (#6031) were purchased from Santa Cruz Biotechnology Inc. (Santa Cruz, CA). Rabbit Ab to pSmad1/5 (41D10)(#9516) was purchased from Cell Signaling Technology (Beverly MA). Rabbit pAb to β-Actin (#A2066) was purchased from Sigma-Aldrich. Mouse mAb to the HA tag (#26183) was from Thermo Fisher Scientific (Rockford, IL). Goat pAb to RTN4/NogoB (N18) (#11027) was purchased from Santa Cruz Biotechnology Inc. (Santa Cruz, CA). Mouse mAbs to EEA1 (#610457), Rab5 (#610724), and LAMP2 (#555803) were purchased from BD Biosciences (Eugene, OR).Respective AlexaFluor 488-, 594- or 645-tagged secondary donkey antibodies to rabbit (#A-11008 and #A-11012), mouse (#A-21202 and #A-21203) or goat (#A-11055 and #A-11058) IgG were from Invitrogen Molecular Probes (Eugene, OR).

### Chemical reagents

Dynamin GTPase inhibitor dynasore (#D7693), microtubule inhibitor nocodazole (#M1404) and microtubule stabilizer paclitaxel (#T7402) were purchased from Sigma-Aldrich (St. Louis, MO).

### Statistical analyses

For luciferase reporter assays, the luciferase activity was presented as mean ± SE after normalization with the β-galactosidase activity among different samples. Statistical significance of such data was evaluated using a two-tailed Student’s t-test in Microsoft Excel software in comparisons between two groups; ANOVA was used to evaluate significance in experiments with multiple groups.
